# Demineralized Dentin Matrix Promotes Bone Regeneration Through IDO1-Mediated Th17/Treg Cell Balance Modulation

**DOI:** 10.1016/j.identj.2025.103853

**Published:** 2025-09-04

**Authors:** Shuyu Zhu, Rongkun Chen, Shu Zhang, Zhiya Wang, Xueyuan Meng, Peng Ling, Jing Zhou, Zhigang Xie

**Affiliations:** a Dept. of Oral Implantology, the Affiliated Stomatology Hospital of Kunming Medical University, Kunming, China; b Yunnan Key Laboratory of Stomatology, School of Stomatology, Kunming Medical University, Kunming, China; c Dept. of Periodontology, the Affiliated Stomatology Hospital of Kunming Medical University, Kunming, China; d Dept. of Orthodontics, Yan’an Hospital Affiliated to Kunming Medical University, Kunming, China; e Yunnan Province Key Laboratory of Tumor Immunological Prevention and Treatment, Yan’an Hospital Affiliated to Kunming Medical University, Kunming 650051, China

**Keywords:** Treg cells, Th17 cells, Demineralised dentin matrix, Indoleamine 2,3-dioxygenase 1, Osteoimmunology, Bone regeneration

## Abstract

**Objectives:**

Demineralised dentin matrix (DDM) is an effective scaffold material for bone tissue engineering. However, the osteoimmunological mechanism of DDM remains unexplored. Th17/Treg cell balance has been noticed as a crucial factor in bone regeneration. The aim of this research is to elucidate the influence of DDM on the balance of Th17/Treg and its underlying molecular mechanisms.

**Methods:**

The study examined DDM’s effects on Th17/Treg cell balance by co-incubating DDM extract with peripheral blood mononuclear cells (PBMCs). Transcriptomics and bioinformatics identified DDM-related differentially expressed genes. A naïve CD4⁺ T cell-bone marrow–derived mesenchymal stem cells (BMSCs) co-culture model assessed DDM’s immunomodulatory effects on Th17/Treg differentiation and their impact on BMSC viability and osteogenic differentiation. *In vivo*, a rabbit mandibular defect model with DDM implantation evaluated bone repair and Th17/Treg regulation.

**Results:**

DDM promoted Treg cell differentiation in PBMCs and increased anti-inflammatory cytokines. Transcriptomic analysis shows that indoleamine 2,3-dioxygenase 1 (IDO1) is a key regulator of Th17/Treg balance. In naïve CD4⁺ T cells, DDM enhanced Treg differentiation, which was blocked by IDO1 inhibition. This immunomodulation improved BMSCs viability and osteogenic differentiation which was attenuated by IDO1 inhibition. Co-culture with naïve CD4⁺ T cells further amplified DDM’s pro-osteogenic effects. In rabbit mandibular defects, DDM enhanced bone repair and was suppressed with IDO1 inhibition.

**Conclusion:**

This study demonstrates that DDM regulates the Th17/Treg cell balance by promoting Treg cell differentiation through IDO1 upregulation, with minimal impact on Th17 cells. This immunomodulatory mechanism is a key factor underlying DDM’s significant role in bone repair.

## Introduction

Bone deficiencies, resulting from injuries, infections, tumours, surgical treatment, metabolic bone diseases and congenital factors, have a significant negative impact on overall well-being. At present, clinical treatments for bone defects include autografts, allografts and xenografts. Autografts represent the preferred clinical option for bone regeneration, owing to their ideal combination of osteogenic potential, osteoconductive matrix and osteoinductive factors, and not provoking immune rejection when implanted. However, given the limited supply of autologous bone and the need to perform additional operations, which increases patients’ discomfort and surgical morbidity, an alternative to avoid these drawbacks is preferred.^1^

Another option for bone lesion repair is the demineralised dentin matrix (DDM), which is derived from autogenous tooth dentin and is produced through a series of processes including demineralisation, rinsing, grinding and sterilisation, among others.^2^ Since the discovery that DDM possesses ectopic osteogenic properties and is able to release bone morphogenetic proteins (BMPs), DDM is gradually gaining popularity as a potential ideal bone graft material.^3^

Clinical studies have reported numerous cases of bone repair by DDM and demonstrated its advantages in bone defect repair, such as in alveolar ridge preservation and guided bone regeneration.^4^, ^5^, ^6^, ^7^, ^8^ However, there are also limitations associated with DDM. Similar to autologous bone, the supply of autologous teeth is limited, and their extraction may cause additional trauma to patients. For allogeneic and xenogeneic teeth, on the other hand, potential ethical concerns (for specific religious) and/or epidemic risks can’t be ruled out. Regardless of autologous, allogeneic or xenogeneic sources, the preparation of DDM is tedious and costly, and thus the product is expensive. All these limitations hamper the wide usage of DDM in the clinic. On the other hand, synthetic materials like hydroxyapatite are cost effective, easy to process, and highly biocompatible, but they exhibit limited capabilities for promoting angiogenesis and osteogenesis.^9^^,^^10^ Therefore, an ideal bone repair material should combine the superior osteogenic properties of biological materials with the advantages of being safe, cheap, free of ethical concerns and easy for industrial production as that of hydroxyapatite. Therefore, delving into the intrinsic mechanisms of the osteogenesis induction effect of DDM may provide supportive information for the development of such an ideal bone repair material.

Arron et al. proposed the concept of osteoimmunology, one discipline that studies the interaction between the immune system and the skeleton system, emphasising the key role of the immune system in bone metabolism.^11^ T-helper 17 cell (Th17) and regulatory T cell (Treg) balance has been noticed as one crucial factor in bone regeneration.^12^ In this research, we aimed to identify the osteoimmunological mechanism of DDM during osteogenesis. We explored how the Th17/Treg cell balance is regulated and the contribution of indoleamine 2,3-dioxygenase 1 (IDO1) to Th17/Treg balance during bone regeneration induced by DDM in cell models and New Zealand rabbits.

## Methods

### Preparation of DDM extract

DDM (GMCB) extract was prepared at a ratio of 100 mg DDM to 1 mL of serum-free DMEM medium. After 5 days of extraction, the supernatant was collected to obtain the DDM extract, which was stored at 4 °C for future use.

### Isolation and culture of peripheral blood mononuclear cells (PBMCs)

The protocol for peripheral blood sample collection was ethically reviewed and approved by the Affiliated Stomatological Hospital of Kunming Medical University (KYKQ2024MEC005). Peripheral blood samples were collected from healthy volunteers using heparin anticoagulant tubes. PBMCs were isolated and purified using Ficoll density gradient centrifugation. Following 48 hours of incubation under the described environment, PBMCs along with the culture medium were gathered for downstream analysis.

### Isolation of naïve CD4^+^ T cells

Naïve CD4^+^ T cells were purified from the PBMCs using the EasySep Human Naïve CD4^+^ T Cell Isolation Kit II (STEMCELL). The PBMCs obtained from whole blood separation (1 × 10^8^ cells/mL) were treated with 10 μL/mL of ECR Blocker, and the sample was transferred to a 5-mL flow tube. The Isolation Cocktail at 50 μL/mL was added, mixed thoroughly, and the mixture was incubated for 7.5 minutes. The Isolation Cocktail contains specific antibody complexes designed to selectively recognise and attach to surface markers expressed by non-target cells.

Next, the Depletion Cocktail at 50 μL/mL was introduced, mixed gently, and allowed 2.5 minutes for incubation. Prior to adding RapidSpheres, they were vortexed for 30 seconds to achieve homogeneous suspension, after which 75 μL/mL of RapidSpheres were incorporated, mixed thoroughly, and incubated for another 2.5 minutes. RapidSpheres are magnetic particles coated with streptavidin, which can bind to biotin, thereby attaching biotin-labelled non-target cells to the magnetic beads. An appropriate amount of culture medium was added to bring the sample volume to 2.5 mL, after which it was gently pipetted 2-3 times, and the flow tube was placed in the EasySep magnet for incubation for 2.5 minutes. Under magnetic separation, bead-conjugated cells accumulated along the tube wall. After incubation, the magnet and flow tube were inverted, and the unlabelled cells (the target naïve CD4⁺ T cells) were carefully poured into a new centrifuge tube for further use.

### Identification of the surface markers and multidirectional differentiation capabilities of bone marrow–derived mesenchymal stem cells (BMSCs)

BMSCs (human, PRI-H-00137, Zqxzbio) were cultured in a standard complete medium, and surface markers CD90(+), CD105(+), CD34(–) and CD45(–) (CD105-PE, CD34-FITC, CD45-PE, CD90-FITC, ThermoFisher) were analysed by flow cytometry.

BMSCs were cultured in osteogenic induction media (Procell), and ALP staining was performed after 14 days. ARS staining was performed after 21 days to assess osteogenic differentiation.

BMSCs were cultured in adipogenic induction media (Procell), and oil red O staining was performed after 21 days to assess adipogenic differentiation.

BMSCs were cultured in chondrogenic induction media (Procell) for 21 days. Alcian Blue staining was performed to evaluate chondrogenic differentiation.

### Experimental setup and co-culture conditions for BMSCs and naïve CD4^+^ T Cells

1. CD4+BMSC: naïve CD4^+^ T cells were plated in the lower compartment of a Transwell plate while BMSCs were plated in the upper compartment. Co-culture was performed in a DMEM medium.
2. DDM+BMSC: BMSCs were plated in the upper compartment of a Transwell plate and cultured in DMEM medium with a DDM extract.
3. CD4+DDM+BMSC: naïve CD4^+^ T cells were plated in the lower compartment of Transwell plate while BMSCs were plated in the upper compartment. Co-culture was performed in a DMEM medium with a DDM extract.
4. CD4+DDM+Inh+BMSC: naïve CD4^+^ T cells were plated in the lower compartment of Transwell plate while BMSCs were plated in the upper compartment. Co-culture was performed in a DMEM medium with a DDM extract and the IDO1 inhibitor 1-Methyltryptophan (1-MT).^13^
5. DDM+Inh+BMSC: BMSCs were plated in the upper compartment of a Transwell plate and cultured in a DMEM medium with a DDM extract and the IDO1 inhibitor 1-MT.

For the CD4+BMSC, CD4+DDM+BMSC and CD4+DDM+Inh+BMSC groups: After 48 hours of co-culture between naïve CD4^+^ T cells and BMSCs, T cells were collected for subsequent experiments. After 14 and 21 days of co-culture, BMSCs from the upper compartment of the Transwell were collected for further detection.

For the DDM+BMSC and DDM+Inh+BMSC groups: After 14 and 21 days of culture with a DDM extract, BMSCs from the upper compartment of the Transwell were collected for subsequent analysis.

### RNA extraction and library preparation

Following 48-hour culture periods, triplicate cell samples from both Control and DDM groups were harvested. RNA isolation was performed with the TRIZOL reagent. Quantitative and qualitative RNA analyses were conducted using spectrophotometric measurement and capillary electrophoresis (Agilent 2100 Bioanalyzer), respectively. For sequencing preparation, the VAHTS Universal V5 RNA-seq Library Prep Kit was used, with strict adherence to the recommended workflow.

### RNA-Seq and DEG detection

Library sequencing was performed using an Illumina Novaseq 6000 system (150 bp paired-end configuration). Initial quality control and adapter trimming of raw fastq files were conducted with fastp,^14^ yielding high-quality reads for subsequent processing. Reference genome alignment was achieved through HISAT2,^15^ followed by FPKM-based expression quantification (Fragments Per Kilobase of transcript per Million mapped reads).^16^ Read counts for each gene were obtained using HTSeq-count.^17^ Principal component analysis and visualisation were performed to evaluate the biological replicates.

DEGs were identified using DESeq2 software,^18^ with genes meeting the threshold of |log_2_^FC^| > 2 and q < 0.05. We employed hierarchical clustering analysis (R, v3.2.0) to explore the expression dynamics of differentially expressed genes across all sample groups. Volcano plots were generated using R packages to visualize up- and downregulated genes. The hypergeometric algorithm was applied to gene ontology (GO) analysis to reveal functionally enriched terms with statistical significance.^19^ Bar plots and chord diagrams of the significantly enriched GO terms were generated using R (v3.2.0).

### Flow cytometry

The collected cells were stimulated with a Cell Stimulation Cocktail. For Th17 cell identification, lymphocytes were stained with anti-CD4 FITC and anti-IL-17A PE antibodies (Bioss). For Treg cell identification, lymphocytes were stained with anti-CD4 FITC, anti-CD25 APC and anti-Foxp3 PE antibodies (Bioss). The percentages of CD4^+^CD25^+^Foxp3^+^ and CD4^+^IL-17A^+^ cells were detected using Novocyte 2060R Flow Cytometer (ACEA).

### WB

The cells were collected into centrifuge tubes and lysed on ice for protein extraction. Stacking and separating gels were prepared, and electrophoresis buffer was added to the electrophoresis tank. Protein lysates were electrophoresed on 10% SDS-polyacrylamide gels and subsequently electroblotted onto PVDF membranes.

The membrane was incubated with pre-prepared primary antibodies at room temperature for 4 hours. The primary antibodies included anti-RORγt, anti-IDO1, anti-osteoprotegerin (OPG), anti-osterix (Osx), antiosteopontin (OPN), anti–runt-related transcription factor 2 (RUNX2), anti-collagen type I (COL I), anti–bone sialoprotein (BSP), anti-osteocalcin (OCN), antireceptor activator of nuclear factor kappa-B ligand (RANKL), anti-receptor activator of nf-kappaB (RANK) (dilution ratio 1:1000, Bioss), anti-Foxp3 (1:1000, Abcam) and anti-GAPDH (1:2000, Zsgb-bio). The anti-GAPDH antibody was used for normalisation. The membrane was incubated with universal secondary antibodies (Abmart) for 1 hour, followed by 3 washes with Tris-Buffered Saline with Tween 20 (TBST) for 5 minutes each. Finally, the membrane was dried with absorbent paper and then visualised using ECL chemiluminescence imaging system (Tanon). The band intensity was analysed using Image J software. The experiment was performed in triplicate.

### RT-qPCR

The total ribonucleic acid of the induced cells was extracted with the riboSCRIPTTM mRNA/lncRNA RT-qPCR Starter Kit (RIBOBIO). RT-qPCR was performed using the LightCycler PCR System with SYBR qPCR Master Mix (Vazyme). The primer sequences (5’-3’) were as follows: Foxp3 (Forward: AAGCAGCGGACACTCAAT, Reverse: AGGTGGCAGGATGGTTTC), GAPDH (Forward: TTGCCCTCAACGACCACTTT, Reverse: TGGTCCAGGGGTCTTACTCC), RORγt (Forward: TTGAGTATAGT-CCAGAAC, Reverse: CACATCTTCCAAGAGTAA), IDO1 (Forward: CCTGACTTATGAGAACAT, Reverse: ATTGCCTTGAATACAGTA), BSP (Forward: CGAGCCTATGAAGATGAG, Reverse: GTGGTGGTAGTAATTCTGA), Col I (Forward: AGAAGAACTGGTACATCA, Reverse: CATACTCGAACTGGAATC), OCN (Forward: CAGAGTCCAGCAAAGGTG, Reverse: AGCCATTGATACAGGTAGC), OPG (Forward: AATGTGGAATAGATGTTACC, Reverse: TCTACCAAGACACTAAGC), OPN (Forward: AATGATGAGAGCAATGAG, Reverse: GTCTACAACCAGCATATC), Osx (Forward: CCCTTTACAAGCACTAAT, Reverse: ATCATTAGCATAGCCTGA), RANKL (Forward: TACAGAGTATCTTCAACTAATG, Reverse: CTCCAGACCGTAACTTAA), RANK (Forward: CCAGTGAGAAGCATTATG, Reverse: CTGTCAGAGGTAGTAGTG), RUNX2 (Forward: ACCATAACCGTCTTCACAA, Reverse: GAGGTCCATCTACTGTAACTT). Using the LightCycler System (Roche), we conducted qPCR analyses. The 2–^ΔΔ^ Ct method was applied to calculate relative expression levels based on obtained Ct values, normalising to GAPDH as the reference gene.

### ELISA

From the manufacturer’s instructions, the concentrations of IL-17, IL-6, TGF-β and IL-10 were measured using detection kits. The ELISA kits used included Human IL-17 ELISA kit, human IL-6 ELISA kit, human TGF-β ELISA kit and human IL-10 ELISA kit (Mlbio) to detect, respectively, the concentrations of IL-17, IL-6, TGF-β and IL-10 *in vitro*. Rabbit IL-17 ELISA kit, rabbit IL-6 ELISA kit, rabbit TGF-β ELISA kit and rabbit IL-10 ELISA kit (Mlbio) were used to detect, respectively, the concentrations of IL-17, IL-6, TGF-β and IL-10 in the peripheral blood of the rabbits. Absorbance readings (450 nm) were acquired using a SpectraMax 190 microplate spectrophotometer (Molecular).

### CCK-8

After 21 days of culture, BMSCs in the CD4+BMSC, DDM+BMSC, CD4+DDM+BMSC, CD4+DDM+Inh+BMSC and DDM+Inh+BMSC groups were collected. Cells (5 × 10³/well) were plated in 96-well plates and maintained at 37 °C / 5% CO₂. Following 24 hours of incubation, the medium was aspirated and replaced with a 100-μL basal medium according to experimental groupings.

CCK-8 reagent (Beyotime) (10 μL) was added per well, followed by 2 hours of incubation (37 °C, dark). Optical density (OD) values, with absorbance at 450 nm, were then quantified using a SpectraMax 190 spectrophotometer. Viability was calculated using the following formula: [(OD experimental – OD blank) / (OD control – OD blank)] × 100%.

### Construction of mandibular defect model and material implantation in New Zealand rabbits

Thirty healthy New Zealand white rabbits were provided by Yikeda Biotechnology Company (Certificate No. SCXK(Qian)2021-0001). Animal experiments were approved by the Institutional Animal Care and Use Committee (IACUC) under approval number PZ20240402. New Zealand rabbits were routinely disinfected and anaesthetised by intravenous injection of 3% sodium pentobarbital (1 mL/kg). After anaesthesia took effect, the rabbits were placed on the operating table, shaved and disinfected. The surgical site was exposed, and the mandibular defect model was prepared according to the following.

Thirty rabbits were randomly allocated to 5 groups:

Sham: After exposing the bone surface, the wound was sutured immediately.
Model (Mod): The bone defect was covered with a collagen membrane (Geistlich Bio-Gide).
Inhibitor: The bone defect was treated with the inhibitor 1-MT (starting 1 day before surgery, the inhibitor was injected intraperitoneally daily at 0.33 mg/kg).^20^^,^^21^
DDM: The bone defect was treated with DDM particles and a collagen membrane.
DDM+Inhibitor: The bone defect was treated with DDM particles and 1-MT and then covered with a collagen membrane.

A flap was incised and reflected 5 mm distal to the first molar of the right mandible to expose the jawbone, with care taken to protect the mental nerve. After exposing the bone surface, a cylindrical critical-sized mandibular bone defect of 8 mm × 3 mm^22^ was created using a high-speed surgical handpiece (KAVO), cooled with sterile saline. Bone debris in the defect area were removed, and the wound was thoroughly rinsed with saline. Materials were implanted by the group, with subsequent collagen membrane coverage of the defect. Haemostasis was ensured, and the wound was sutured. Postoperatively, intramuscular ampicillin (65,000 units/kg/day) was administered for 3 successive days as anti-infection prophylaxis. The rabbits were raised normally until sample collection.

Blood samples were collected at 1, 4 and 8 weeks postoperatively, and serum was isolated and tested using ELISA kits. Three rabbits from each group were randomly selected for euthanasia at 1 and 8 weeks postoperatively, and their mandibles were collected for subsequent experiments.

### Micro-CT

At 8 weeks postoperatively, rabbits were euthanised, and dissection was performed to harvest tissue samples. After a 24-hour fixation in 4% paraformaldehyde, specimens were scanned (Bruker Skyscan 1276, Kontich) following hydroxyapatite phantom calibration. Reconstruction (NRecon v. 1.7.4.2) and 3-dimensional rendering (CTvox v. 3.3.0) preceded quantitative analysis (CT Analyzer v. 1.20.3.0) of bone mineral density (BMD), bone volume fraction (BV/TV), and trabecular thickness (Tb.Th) in the selected region of interest (ROI).

### Tissue sample processing and section preparation

Following a 7-day fixation in 10% neutral buffered formalin, tissue specimens underwent standard histological processing comprising decalcification, graded ethanol dehydration, xylene clearing, paraffin embedding and microtome sectioning at 5-μm thickness prior to slide mounting and baking.

### H&E staining

Following rehydration, tissue sections were sequentially treated with Hematoxylin (Sigma), water rinse, 1% hydrochloric acid, water wash and eosin (Beyotime). The stained sections then underwent graded ethanol dehydration, xylene clearing and coverslipping. Slides were observed and photographed using a Pannoramic MIDI holographic scanner (3DHISTECH).

### ALP staining

Paraffin-embedded sections were dewaxed and rehydrated with xylene, then incubated in ALP staining solution (Solarbio) at room temperature for 20 minutes. ALP activity generated a coloured precipitate upon reaction with the substrate. The sections were rinsed with water to stop the reaction and stained with haematoxylin for nuclear counterstaining. The stained sections then underwent graded ethanol dehydration, xylene clearing and coverslipping. Slides were observed and photographed using a Pannoramic MIDI holographic scanner. Image Pro Plus 6.0 was used to measure the greyscale values of the stained regions and calculate the average greyscale value as a quantitative indicator.

### ARS staining

Paraffin-embedded sections were subjected to dewaxing and rehydration using xylene. The fixed sections or cellular samples were then incubated in a 2% ARS staining solution (Solarbio) for 30 minutes. After discarding the H&E staining solution, the sections were rinsed with deionised water until no residual dye was present. Finally, the sections were observed and photographed using the Pannoramic MIDI holographic scanner.

### Masson staining

According to the protocol of the Masson Trichrome Staining Kit (Solarbio), paraffin-embedded sections were dewaxed and rehydrated using xylene. Reagents A1 and A2 for Weigert's iron hematoxylin staining were mixed, and the sample sections were washed, differentiated, and counterstained with Masson's Bluing for 5 minutes. They were then stained with fuchsin-eosin and treated with phosphomolybdic acid and aniline blue. Finally they were dehydrate with ethanol, cleared with xylene, mounted, and scanned for analysis.

### IHC

Paraffin-embedded sections were dewaxed, rehydrated and subjected to antigen retrieval in a citrate buffer. After PBS washes, sections were encircled with a hydrophobic pen and blocked with 5% goat serum for 1 hour. Primary antibodies (anti-Osx, anti-BSP, anti-OCN, anti-IDO1, anti-TDO2, anti-RANKL, anti-RANK) were applied and incubated overnight at 4 °C. After PBS washes, endogenous peroxidase was blocked for 30 minutes, followed by a 20-minute incubation with a reaction enhancer. The sections were then incubated with HRP-linked secondary antibodies. DAB chromogen was used for staining development, terminated with distilled water and counterstained with haematoxylin. Sections were treated with 1% HCl-ethanol, rinsed, dried at 60 °C, cleared in xylene and mounted. Slides were scanned using a Pannoramic MIDI holographic scanner.

### IF

Tissue sections underwent PBS washing and microwave-assisted (4 minutes at medium power, 5 minutes at low power) antigen retrieval using EDTA buffer (pH 9.0). Sections were washed again, dried and encircled with a hydrophobic pen. Goat serum (5%) was used for 1 hour of blocking. Primary antibodies (anti-RORγt, anti-Foxp3, anti-OPG, anti-OPN, anti-RUNX2, anti-COL I) were applied at 4 °C overnight. Secondary antibodies were applied for 60 minutes. Nuclei were counterstained with DAPI.

### Statistical analysis

The data were analysed statistically using GraphPad Prism 9.0 software and are presented as mean values plus or minus standard deviations. Data meeting normality and homogeneity of variance assumptions were compared between 2 groups using independent *t*-tests, whereas one-way ANOVA was used for comparisons across multiple groups. Data that did not meet the assumptions were evaluated using non-parametric tests. *P < .05; **P < .01; ***P < .001; ****P < .0001; ns, not statistically significant.

## Results

### DDM extract affects Th17/Treg balance by promoting Treg cells differentiation with less effect on Th17 cells in PBMCs

**Fig. 1:**
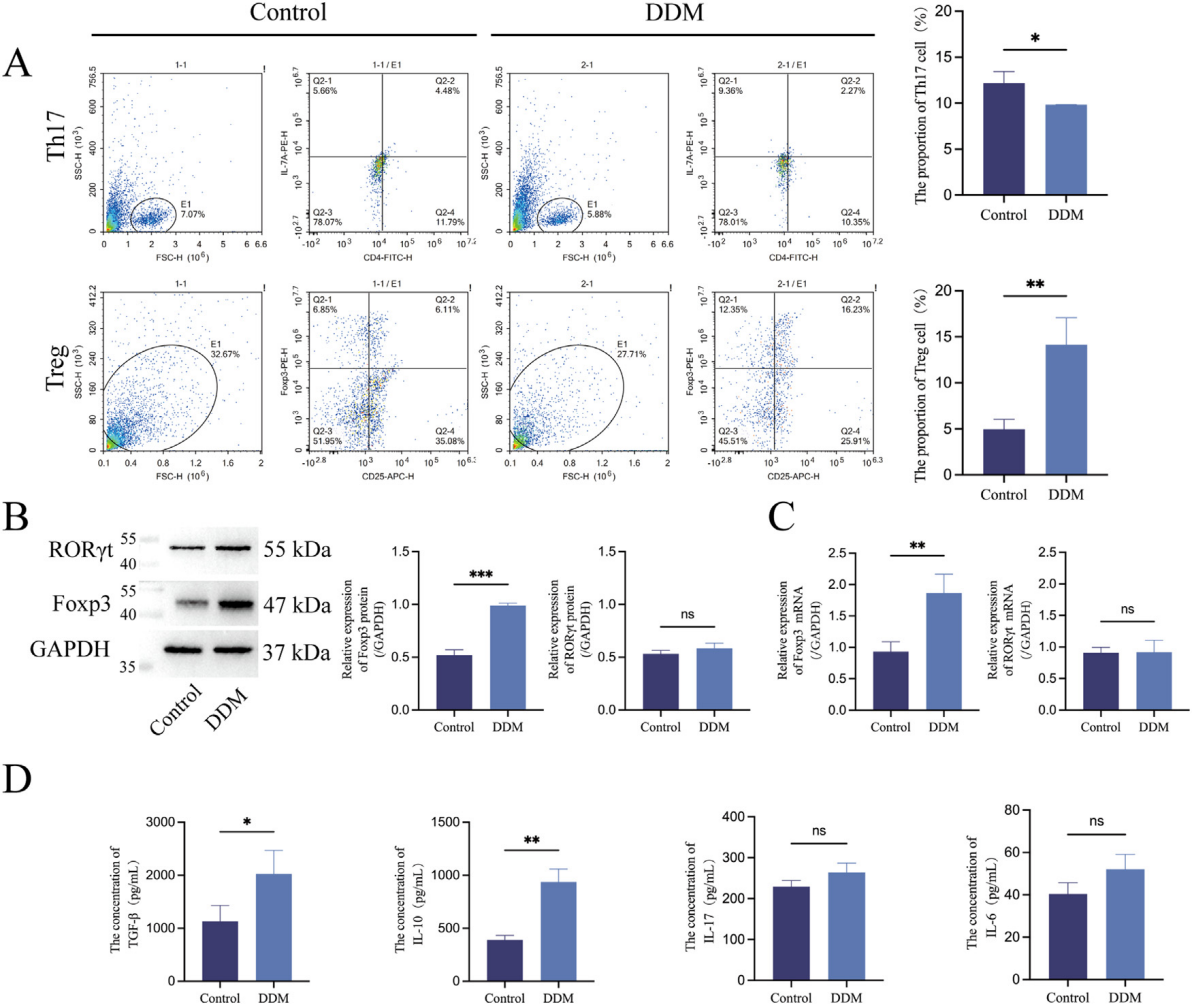
DDM extract affects Th17/Treg balance by promoting Treg cell differentiation while having virtually no effect on Th17 cells in PBMCs. A, Flow cytometry results show that the DDM extract increased the proportion of Treg cells (CD4^+^CD25^+^Foxp3^+^) in PBMCs while the proportion of Th17 cells (CD4^+^IL-17A^+^) slightly decreased. Both WB (B) and RT-qPCR (C) results demonstrated that the DDM extract significantly enhanced the expression of the Treg cell marker Foxp3, whereas the expression of the Th17 cell-specific transcription factor RORγt was virtually unaffected in PBMCs. D, ELISA results revealed a significant increase of TGF-β and IL-10 in the DDM extract group compared to the control while the change in IL-6 and IL-17 associated with Th17 cells was insignificant. Control, culture of PBMSCs without a DDM extract; DDM, culture of PBMCs with a DDM extract.

To assess the effects of DDM on Th17/Treg balance, PBMCs were treated with a DDM extract and then examined using flow cytometry and an immunofluorescence stain of the cell surface marker CD4^+^IL-17A^+^(Th17) and CD4^+^CD25^+^Foxp3^+^ (Treg). It was found that the DDM extract caused the proportion of Treg cells to significantly increase while the proportion of Th17 cells slightly decreased (Figure 1A). These observations suggest that DDM may promote T-cell differentiation toward Treg cells and may affect Th17 cell differentiation less.

To verify this hypothesis, after treatment of PBMCs with a DDM extract, we examined the expression of T-cell molecular markers at both the protein and mRNA levels. Specifically, WB and RT-qPCR experiments were applied to DDM extract–treated PBMCs for the Treg cell marker Foxp3 and the Th17 cell-specific transcription factor RORγt. The WB results showed that, on the protein level, the DDM extract significantly enhanced the expression of Foxp3 expression, whereas the expression of RORγt was virtually unaffected by DDM extracts (Figure 1B). The RT-qPCR showed concordant results with WB (Figure 1C). These results verified that DDM affects Th17/Treg balance by boosting Treg differentiation while showing virtually no effect on Th17 cell differentiation.

To further confirm the above observation on a functional level, the anti-inflammatory cytokines TGF-β and IL-10 associated with Treg cell, and pro-inflammatory cytokines IL-17 and IL-6 associated with Th17 cells were examined using the supernatants of PBMCs cultured by ELISA. ELISA results revealed a significant increase of TGF-β and IL-10 in the DDM extract group compared to the control, whereas the change in IL-6 and IL-17 was insignificant (Figure 1D).

In summary, all the experiments conducted on cellular, protein, mRNA, and function levels demonstrated that a DDM extract affects Th17/Treg balance by promoting Treg cell differentiation while having less effect on Th17 cells in PBMCs.

### Next-Generation Sequencing (NGS)-based transcriptomic assays and subsequent informative analysis suggested that the Th17/Treg balance under the influence of DDM may be mediated by IDO1

**Fig. 2:**
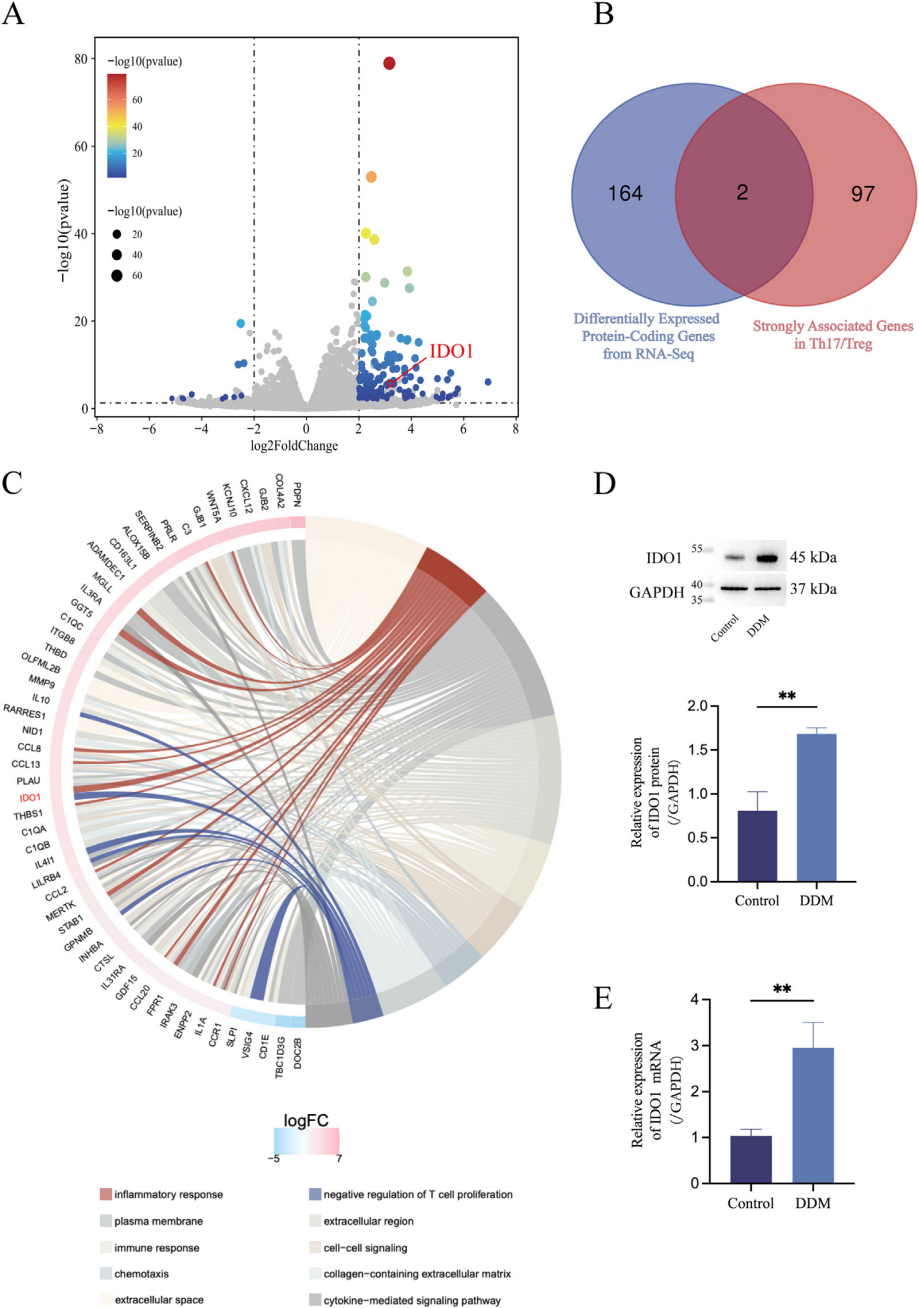
NGS-based transcriptomic assays and subsequent informative analysis suggest that the Th17/Treg balance under the influence of DDM may be mediated by IDO1. IDO1 plays a more dominant role in the DDM-mediated differentiation of PBMCs into Th17/Treg cells. A, The volcano plot for DEGs filtered by the criteria |log_2_^FC^| > 2 and *q* < 0.05. B, Venn diagram revealed IDO1 and IL-10 are key DEGs influenced by DDM in PBMCs, mediating its effects on Th17/Treg cell differentiation. C, Chord diagram of the GO enrichment analysis. On the left are the 10 genes with the greatest |log_2_^FC^| in each classification. On the right it reflects the composition of the classifications. The lines in the middle indicate the correspondence between the classifications and the genes. The external heat map represents the log_2_^FC^ values of the corresponding genes. Among the identified genes, IDO1 was enriched in the functional categories of inflammatory response and negative regulation of T-cell proliferation. D, WB results indicated that the DDM extract significantly enhanced the expression levels of IDO1 in PBMCs. E, RT-qPCR results indicated that the DDM extract significantly enhanced the expression levels of IDO1 in PBMCs. Control, culture of PBMSCs without a DDM extract; DDM, culture of PBMCs with a DDM extract.

To delve into the intrinsic mechanisms by which DDM influences the Th17/Treg balance, PBMCs treated with or without (as control) DDM extracts were collected in triplicate, and the transcriptomic assays were performed by NGS. There were 22,635 genes detected in all samples (Control 1: 19,174, Control 2: 19,080, Control 3: 19,315, DDM 1: 19,480, DDM 2: 19,678, DDM 3: 19,321). Compared to the Control group, 604 differentially expressed genes (DEGs) were selected according to the criteria of |log_2_^FC^| > 1 and *q* < 0.05. When stricter thresholds of |log_2_^FC^| > 2 and *q* < 0.05 were applied, 166 significant DEGs were produced (Figure 2A).

To identify DEGs associated with Th17/Treg cell differentiation, we retrieved genes related to Th17/Treg cells differentiation from the GeneCards database, and 99 strongly associated genes were identified using a Relevance score > 7.^23^ Among the 99 genes, there were only 2 genes, namely IDO1 and IL-10, overlapping with 166 DEGs (Figure 2B), which implies their functional role in DDM’s biological effect.

To further evaluate the functional relevance of these two candidates, we performed GO enrichment analysis on the 166 DEGs. The chord diagram presented the 10 classifications with the smallest *P* and the 10 genes with the largest |log_2_^FC^| in each classification. Among the identified genes, IDO1 was significantly associated with the functional categories of inflammatory response and negative regulation of T-cell proliferation (Figure 2C). These functions align closely with the known roles of Th17 and Treg cells in immune regulation and inflammation. IL-10 also showed in the GO enrichment analysis that it was enriched in categories of negative regulation of T-cell proliferation, extracellular space and extracellular region. It is well known that IL-10 is a cytokine secreted by Treg cells, thus its upregulation is likely a result of enhanced Treg cell proliferation rather than a causal factor for Treg cell proliferation enhancement. Therefore, in subsequent studies, we focused on the IDO1 gene to observe its potential in mediating DDM’s effects on Th17/Treg cell balance.

IDO1 is a rate-limiting enzyme that participates in the tryptophan metabolic process, whereas tryptophan is involved in modulating a number of Tregs and maintaining local immune homeostasis.^24^ After tryptophan is converted to kynurenine, it interacts with the aryl hydrocarbon receptor and plays a key role in inflammation and immune regulation. This mechanistic link further supports the selection of IDO1 as a promising candidate for further investigation.

To verify the results of transcriptome sequencing, WB (Figure 2D) and RT-qPCR (Figure 2E) experiments were performed for the IDO1 gene in DDM-treated PBMCs. The experiments results showed that the IDO1 gene was indeed induced in PBMC at both the mRNA and protein level upon treatment with DDM.

### DDM extract modulates the Th17/Treg balance by upregulating IDO1 to promote Treg cells differentiation while minimally affecting Th17 in the co-culture of naïve CD4^+^ T cells and BMSCs

To verify that DDM influences the Th17/Treg balance via IDO1, a widely used IDO1 inhibitor, 1-MT was used in this study. 1-MT is often used as an IDO1 inhibitor in immune regulation and tumour immune evasion studies because it competitively binds to the active site of IDO1, preventing tryptophan from entering the enzyme’s catalytic center.^25^ Through its IDO1 inhibition activity, 1-MT leads to a decrease in intracellular kynurenine levels, which subsequently affects the activation of the aryl hydrocarbon receptor (AhR). Since AhR activation and IDO1 expression are regulated by a feedback mechanism, the 1-MT-caused kynurenine reduction results in decreased IDO1 expression.^26^, ^27^, ^28^

To investigate the osteogenesis effect of IDO1-mediated Th17/Treg balance alteration, a co-culture system of naïve CD4^+^ T cells and BMSCs was employed in this research. For the co-culture, naïve CD4^+^ T cells were plated in the lower compartment of a Transwell insert while BMSCs were placed in the upper compartment. Then DDM extract with or without IDO1 inhibitor was added to the co-culture system. The differentiation of naïve CD4^+^ T cells was analysed after 48 hours of co-culture, while the osteogenic effect of BMSCs was assessed after 14 or 21 days of co-culture.

**Fig. 3:**
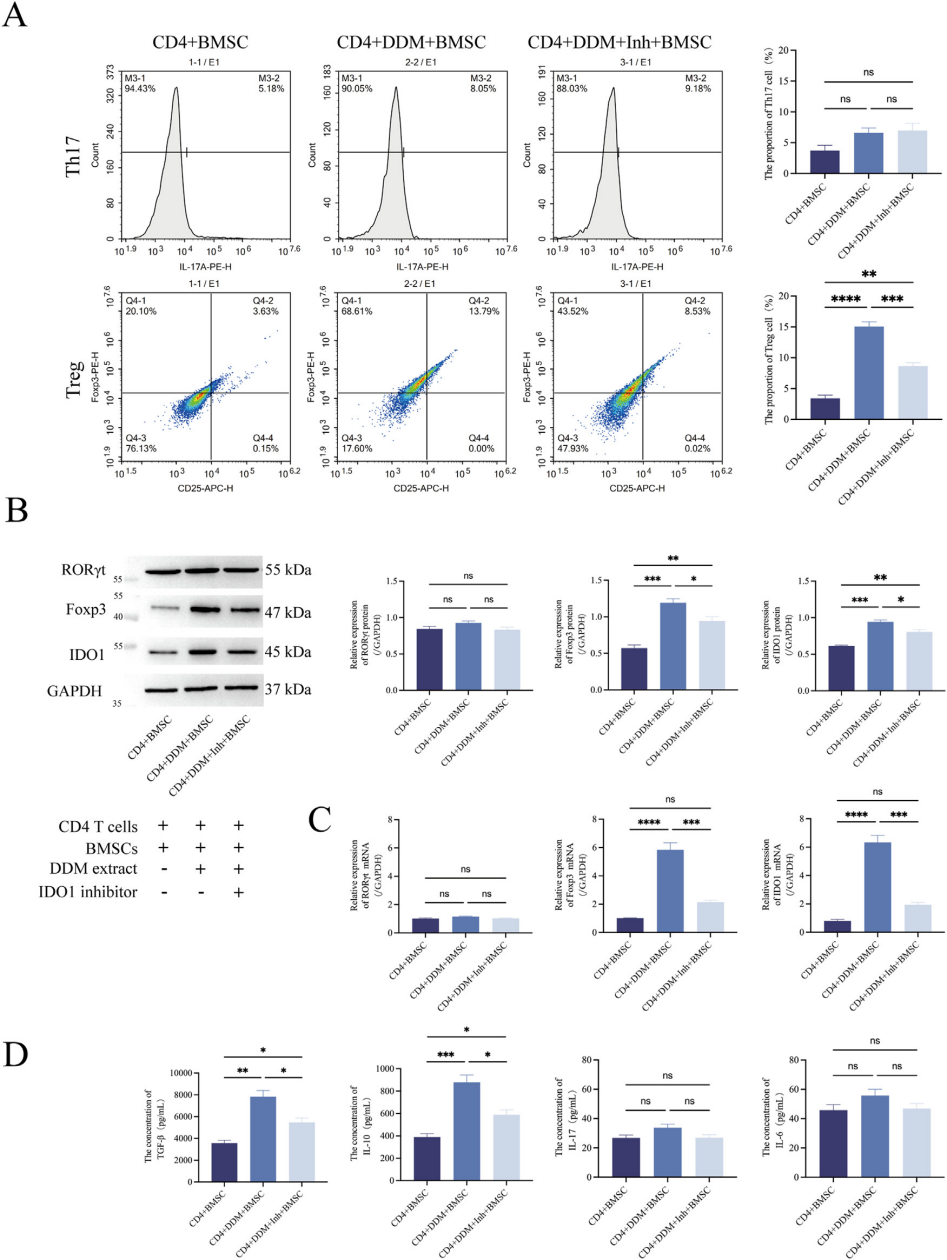
A DDM extract modulates the Th17/Treg balance by activating the IDO1 to promote Treg cell differentiation while minimally affecting Th17 in the co-culture of naïve CD4^+^ T cells and BMSCs. A, Flow cytometry results revealed that the DDM extract increased Treg cell proportion, which dropped significantly with the DDM+IDO1 inhibitor. It also slightly raised Th17 cells, though not significantly, with no notable changes after inhibitor treatment. WB (B) and RT-qPCR (C) results showed that the DDM extract significantly increased IDO1 and Foxp3 expression, which decreased notably with the DDM+IDO1 inhibitor. No significant effects were observed on RORγt mRNA or protein levels. D, ELISA results demonstrated that the DDM extract significantly raised TGF-β and IL-10, which decreased notably with the DDM+IDO1 inhibitor. IL-6 and IL-17 showed no significant changes. CD4+BMSC, co-culture of naïve CD4^+^ T cells and BMSCs; CD4+DDM+BMSC, co-culture of naïve CD4^+^ T cells and BMSCs with the DDM extract; CD4+DDM+Inhibitor (Inh)+BMSC, co-culture of naïve CD4^+^ T cells and BMSCs with the DDM extract and IDO1 inhibitor.

The flow cytometry assay with immunofluorescence stain of cell surface marker revealed that the proportion of Treg cells was significantly augmented by the DDM extract, and this augmentation effect was attenuated by the specific inhibitor of IDO1 (Figure 3A). Meanwhile, the DDM extract slightly elevated the proportion of Th17 cells with no statistical significance. These observations suggest that DDM may modulate the Th17/Treg balance by upregulating IDO1 to promote the differentiation of Treg cells while affecting Th17 only slightly.

WB (Figure 3B), RT-qPCR (Figure 3C) and ELISA experiments (Figure 3D) further confirmed these findings: the DDM extract upregulated IDO1 and Foxp3 expression, increased TGF-β and IL-10 cytokines, and had no significant effect on RORγt and IL-6 and IL-17 cytokines, consistent with flow cytometry results.

In summary, all the aforementioned experiments on cellular, protein, mRNA and function levels demonstrated that a DDM extract modulates the Th17/Treg balance by upregulating IDO1 to promote the differentiation of Treg cells while having virtually no effect on Th17 cells in the co-culture of naïve CD4^+^ T cells and BMSCs.

### DDM extract modulates the Th17/Treg balance via IDO1 to enhance BMSCs viability and osteogenic differentiation in the co-culture system of naïve CD4^+^ T cells and BMSCs

The aforementioned co-culture system was also used to investigate the influence of the IDO- mediated Th17/Treg balance alteration on the osteogenic differentiation of BMSCs. The BMSCs used in the study exhibited typical stem cell surface markers (CD90^+^/CD105^+^, CD45^−^/CD34^−^) and demonstrated multidirectional differentiation potential, including osteogenic, adipogenic and chondrogenic capacities, confirming their identity as functional BMSCs (Supplemental Figure 1).

**Fig. 4:**
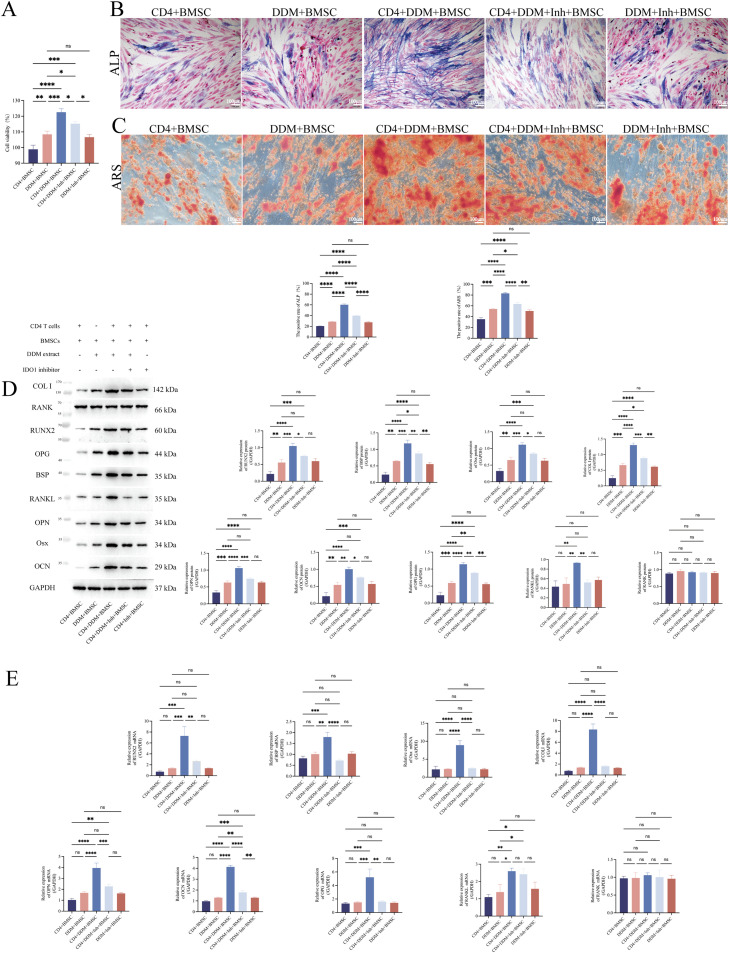
The DDM extract promotes Treg cells differentiation via IDO1 to enhance BMSCs viability and osteogenic differentiation in the co-culture system of naïve CD4^+^ T cells and BMSCs. A, CCK-8 results showed that the DDM extract significantly increased BMSCs viability, which decreased with the IDO1 inhibitor. No significant difference was observed between the Control and DDM+Inhibitor groups. B, Azide-Azide coupling method for ALP staining, with the red arrow indicating the blue precipitate after staining. ALP content significantly increased with the DDM extract but decreased with the DDM+inhibitor treatment compared to the Control. 100X, 1cm:100µm. C, ARS staining, with the white arrow indicating the red calcium nodules after staining. Calcium nodules significantly increased with the DDM extract but decreased with the DDM+inhibitor treatment compared to the Control. 100X, 1cm:100µm. WB (D) and RT-qPCR (E) results indicated that the DDM extract significantly upregulated RUNX2, BSP, Osx, COL I, OPN, OCN, OPG and RANKL. With the DDM+IDO1 inhibitor, RUNX2, BSP, Osx, COL I, OPN, OCN and OPG decreased significantly while RANKL remained unchanged. Neither DDM nor DDM+inhibitor affected RANK expression. CD4+BMSC, co-culture of naïve CD4^+^ T cells and BMSCs; DDM+BMSC, culture of BMSCs with the DDM extract; CD4+DDM+BMSC, co-culture of naïve CD4^+^ T cells and BMSCs with the DDM extract; CD4+DDM+Inh+BMSC, co-culture of naïve CD4^+^ T cells and BMSCs with the DDM extract and IDO1 inhibitor; DDM+Inh+BMSC, culture of BMSCs with the DDM extract and IDO1 inhibitor.

After 21 days of co-culture, the cell viability of BMSCs was assessed using the CCK-8 assay. The results, as depicted in Figure 4A, indicated that in the co-culture system of naïve CD4^+^ T cells and BMSCs, the cell viability and proliferation of BMSCs were significantly augmented by the DDM extract. Notably, the presence of naïve CD4^+^ T cells further enhanced the promoting effects of the DDM extract on cell viability and proliferation of BMSCs. Meanwhile, this augmentation effect was attenuated by an IDO1 inhibitor in a CD4^+^ T cells–dependent manner. These results, together with the aforementioned data (Figure 3), demonstrate that the enhanced viability and proliferation of BMSCs are indeed caused by IDO1-mediated Treg-orientated differentiation of naïve CD4^+^ T cells.

Besides cell viability and proliferation, we also investigated the osteogenic differentiation of BMSCs under the influence of the DDM extract. ALP staining and ARS staining were carried out to detect the early osteogenic marker (ALP activity) and late-stage mineralisation nodule formation in BMSCs, respectively. The results, as depicted in Fig. 4, Fig. 4, indicated that the ALP activity and mineralisation nodule were significantly augmented by the DDM extract in the presence of naïve CD4^+^ T cells, and this augmentation effect was attenuated by IDO1 inhibitor in a CD4^+^ T cell–dependent manner.

To confirm that the presence of naïve CD4^+^ T cells further enhanced the promoting effects of the DDM extract on the osteogenic differentiation of BMSCs, we collected BMSCs after 21 days of co-culture and examined the expression of representative osteogenesis-related factors at the protein (Figure 4D) and molecular (Figure 4E) levels for each stage.

As we know, RUNX2, BSP and Osx represent osteoblast differentiation and proliferation. COL I, OPN and OCN represent bone matrix synthesis and mineralisation. OPG binds to RANKL, inhibiting the interaction between RANKL and RANK, preventing the differentiation and activation of osteoclasts, and is involved in the entire process of bone metabolism.^29^

Both WB (Figure 4D) and RT-qPCR (Figure 4E) results demonstrated that the expression levels of osteogenesis-related factors, representing various stages of bone metabolism (RUNX2, BSP, Osx, COL I, OPN, OCN and OPG), exhibited an accordant trend, namely the expression levels of these osteogenesis-related factors were significantly augmented by the DDM extract in the presence of naïve CD4^+^ T cells, attenuated by the IDO1 inhibitor in a CD4^+^ T cell–dependent manner.

Regarding key regulatory factors of osteoclast differentiation and activation, in the co-culture system of naïve CD4^+^ T cells and BMSCs, the expression level of RANKL was significantly augmented by the DDM extract, and this augmentation effect was attenuated by the IDO1 inhibitor. The expression level of RANK showed no significant differences among the groups.

In summary, within the co-culture system of naïve CD4^+^ T cells and BMSCs, DDM significantly enhanced the cell viability and osteogenic differentiation capacity of BMSCs. This promoting effect was partially dependent on the immunomodulatory effect of DDM. Specifically, the mechanism may be attributed to the upregulation of IDO1 by DDM, which subsequently affects naïve CD4^+^ T cells into Treg cells.

### DDM promotes mandibular bone defect repair via IDO1 in New Zealand rabbits

Our work in this study identified that a DDM extract modulates the Th17/Treg balance by upregulating IDO1 to promote Treg cell differentiation while affecting Th17 to a lesser degree. Meanwhile, this immunomodulatory action of DDM can enhance the viability and osteogenic differentiation capacity of BMSCs. We further investigated whether DDM can effectively promote the repair of mandibular bone defects in rabbits, and if it does, whether its intrinsic mechanism may involve upregulating IDO1 to enhance Treg cell differentiation, thereby regulating the Th17/Treg cell balance.

We constructed mandibular defects in New Zealand rabbits and administered DDM with or without IDO1 inhibitors to observe the repair efficacy of mandibular defects and alterations in the Th17/Treg cell balance.

During the postoperative observation period, the rabbits regained consciousness smoothly and exhibited good activities, appetite, and mental status and no obvious abnormalities. The surgical area healed favourably, with no signs of infection such as redness, swelling or suppuration.

**Fig. 5:**
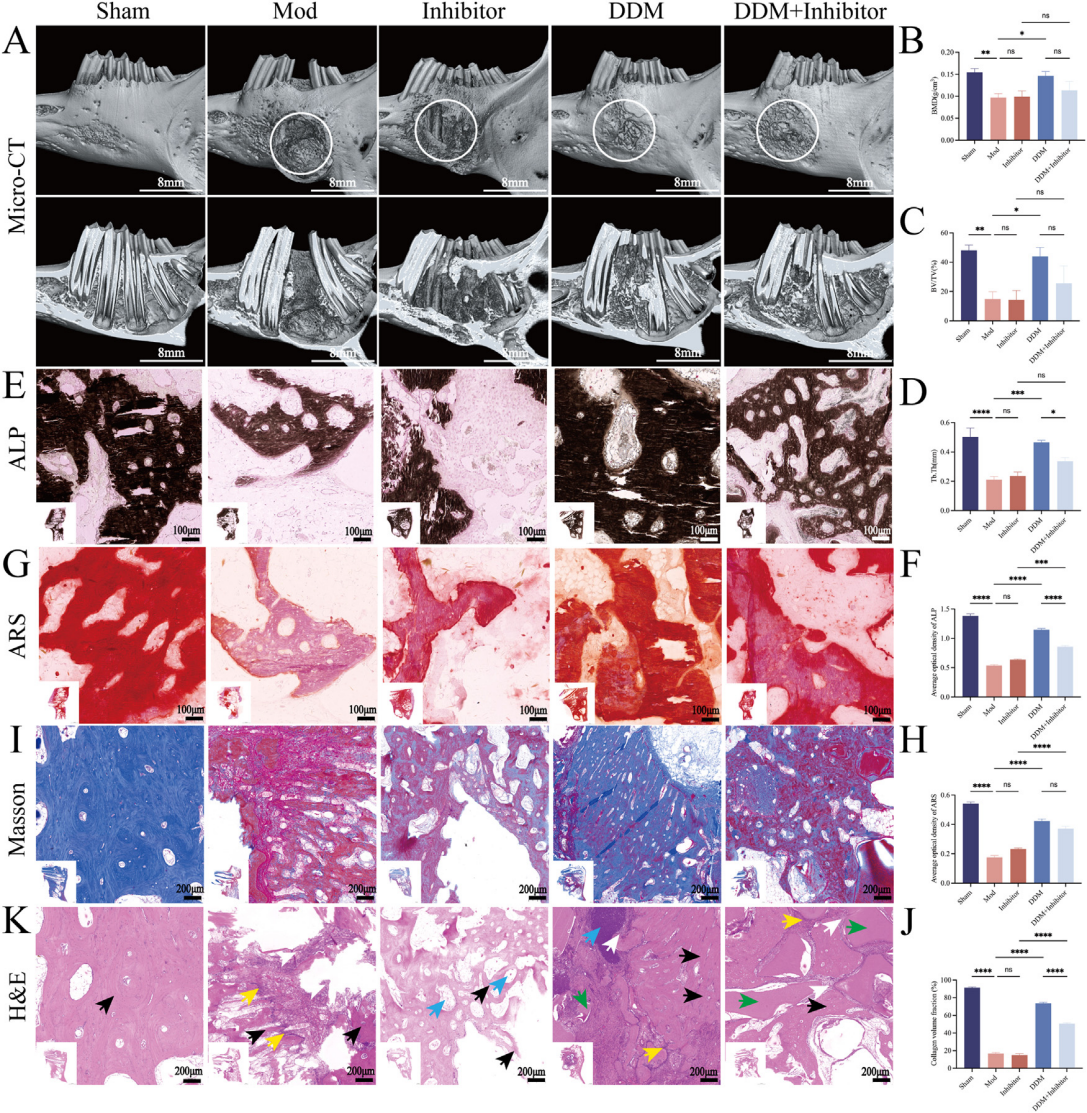
DDM promotes mandibular bone defect repair via IDO1 in New Zealand rabbits at 8 weeks postoperatively. A-D, Three-dimensional reconstruction results show that the DDM group has the best bone repair effect compared to the Mod group, while DDM+inhibitor group shows mediocre repair (A). Quantitative analysis revealed that DDM significantly increased BMD, BV/TV and Tb.Th compared to the Mod group. However, DDM+inhibitor reduced BMD, BV/TV and Tb.Th to some extent compared to DDM alone. 1 cm:8 mm. E-F, The calcium-cobalt method for ALP staining indicates the black precipitate after staining. Local image: 100X, 1 cm:100 µm. Full-field image: 4X, 1 cm:2,000 µm. G-H, ARS staining indicates the red calcium nodules after staining. Local image: 100X, 1 cm:100 µm. Full-field image: 4X, 1 cm:2,000 µm. I-J, Masson staining indicates blue collagen fibres after staining. Local image: 50X, 1 cm:200 µm. Full-field image: 4X, 1 cm:2,000 µm. K, Observation of osteogenesis and inflammation by H&E staining. The black arrow indicates the bone matrix/trabeculae, the white arrow indicates the fibrous connective tissue, the blue arrow indicates the bone marrow, the yellow arrow indicates the inflammatory cells, and the green arrow indicates the residual DDM. Local image: 50X, 1 cm:200 µm. Full-field image: 4X, 1 cm:2,000 µm. Sham, bone exposed without defect creation; Mod, defect without DDM; Inhibitor, defect treated with an IDO1 inhibitor (without DDM); DDM, defect treated with DDM; DDM+Inhibitor, defect treated with DDM and IDO1 inhibitor.

To evaluate the repair of the bone defect area at 8 weeks postoperatively, the mandibles of rabbits were harvested after euthanasia and underwent micro-CT scanning. The 3-dimensional reconstruction outcomes demonstrated that, compared with the Mod group, the bone repair effect in the DDM group was the most optimal, while in the group concurrently administered DDM and the inhibitor, the bone defect repair was mediocre (Figure 5A). The quantitative analysis results (Figure 5B-D) indicated that, in contrast to the Mod group, DDM significantly elevated BMD, BV/TV and Tb.Th. This finding thoroughly substantiates the active role of DDM in promoting new bone formation, enhancing bone density, increasing the proportion of new bone in the total volume and causing the trabecular structure to become thicker. Nevertheless, when IDO1 inhibitors were used in combination with DDM, the values of BMD, BV/TV and Tb.Th decreased to some extent compared to the DDM group, revealing the attenuating effect of IDO1 inhibitors on the osteogenic action of DDM. These observations lead to a hypothesis that DDM can promote mandibular defect bone regeneration in rabbits by upregulating IDO1.

To verify this hypothesis, we further investigated the microstructure details of bone tissue, cellular activity status and specific biological alterations during the tissue repair process subsequent to DDM bone grafting. Histological analyses were performed on sectioned samples using ALP staining (Figure 5E-F), ARS staining (Figure 5G-H), Masson staining (Figure 5I-J) and H&E staining (Figure 5K). ALP staining, ARS staining and Masson staining results demonstrated that the DDM group significantly enhanced ALP expression, increased calcium nodule formation, and elevated collagen content, reflecting improvements in early osteogenic marker activity, late mineralisation, and collagen fibre deposition. However, these effects were attenuated when IDO1 inhibitors were co-administered.

H&E staining results (Figure 5K) indicated that, compared to the Sham group – where the bone matrix (black arrow) exhibited a lamellar, orderly structure without defects or inflammation – the other four groups displayed varying degrees of structural abnormalities and damage. In the DDM-treated groups, jawbone injuries were significantly alleviated compared to the Mod group, with increased osteoid tissue (black arrow), greater trabecular bone quantity and distinct bone marrow (blue arrow). Additionally, fibrous connective tissue decreased (white arrow) while residual DDM (green arrow) and inflammatory cell infiltration (yellow arrow) were observed. Compared to the DDM+Inhibitor group, the DDM group showed better integration of the bone graft material with surrounding tissues, forming more compact osteoid tissue. The Inhibitor group exhibited injury levels similar to the Mod group, with abnormal bone structure, enlarged interspaces, reduced trabecular bone quantity, thinner trabeculae (black arrow) and bone marrow deficiency (blue arrow).

**Fig. 6:**
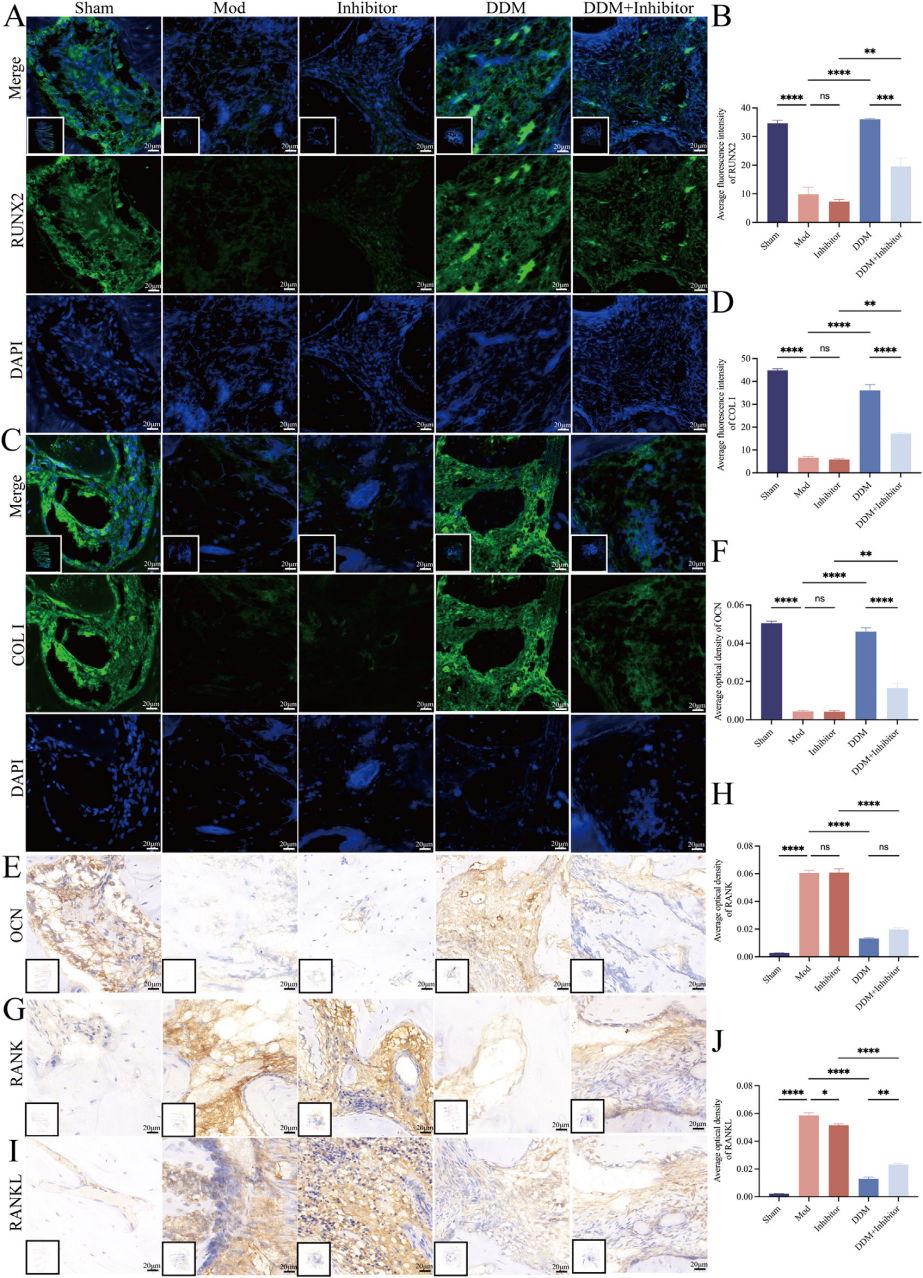
DDM promotes the expression of osteogenesis-related factors while inhibiting key regulators of osteoclast differentiation via IDO1 in New Zealand rabbits. IF (A-D) and IHC (E-J) results demonstrated that compared to the Mod group, the DDM group significantly increased RUNX2 (A-B), COL I (C-D) and OCN (E-F) expression, which was reduced by the IDO1 inhibitor. Additionally, the DDM group decreased RANK (G-H) and RANKL (I-J) expression, but this effect was reversed by the IDO1 inhibitor. Local image: 400X, 1 cm:20 μm. Full-field image: 4X, 1 cm:2,000 μm. Sham, bone exposed without defect creation; Mod, defect without DDM; Inhibitor, defect treated with an IDO1 inhibitor (without DDM); DDM: defect treated with DDM; DDM+Inhibitor, Defect treated with DDM and IDO1 inhibitor.

To further elucidate the molecular mechanisms, IHC and IF were used to examine the expression of osteogenesis-related factors (RUNX2, BSP, Osx, COL I, OPN, OCN, OPG) and key regulators of osteoclast differentiation and activation, RANK and RANKL, in mandibular repair tissue at 8 weeks post-surgery. The results (Figure 6 shows RUNX2, COL I, OCN, RANK and RANKL; other osteogenesis-related factors are presented in Supplementary Figure 2) revealed that, compared to the Mod group, the DDM group significantly upregulated the expression of osteogenesis-related factors. However, co-administration of the IDO1 inhibitor markedly reduced the expression of these proteins. Simultaneously, the DDM group significantly downregulated RANKL and RANK expression compared to the Mod group, but this effect was reversed when the IDO1 inhibitor was added. The *in vivo* effects of DDM on RANKL and RANK may be partially attributed to its promotion of Treg cells and TGF-β and IL-10, which mitigate inflammation and restore the balance between osteogenesis and osteoclastogenesis in the defect area.^30^

In summary, all the above experiments demonstrated that DDM promotes mandibular bone defect repair in New Zealand rabbits. These osteogenetic outcomes were suppressed following treatment with an IDO1 inhibitor.

### DDM upregulates IDO1 to modulate the Th17/Treg cell balance and improve the inflammatory environment during bone defect repair in New Zealand rabbits

**Fig. 7:**
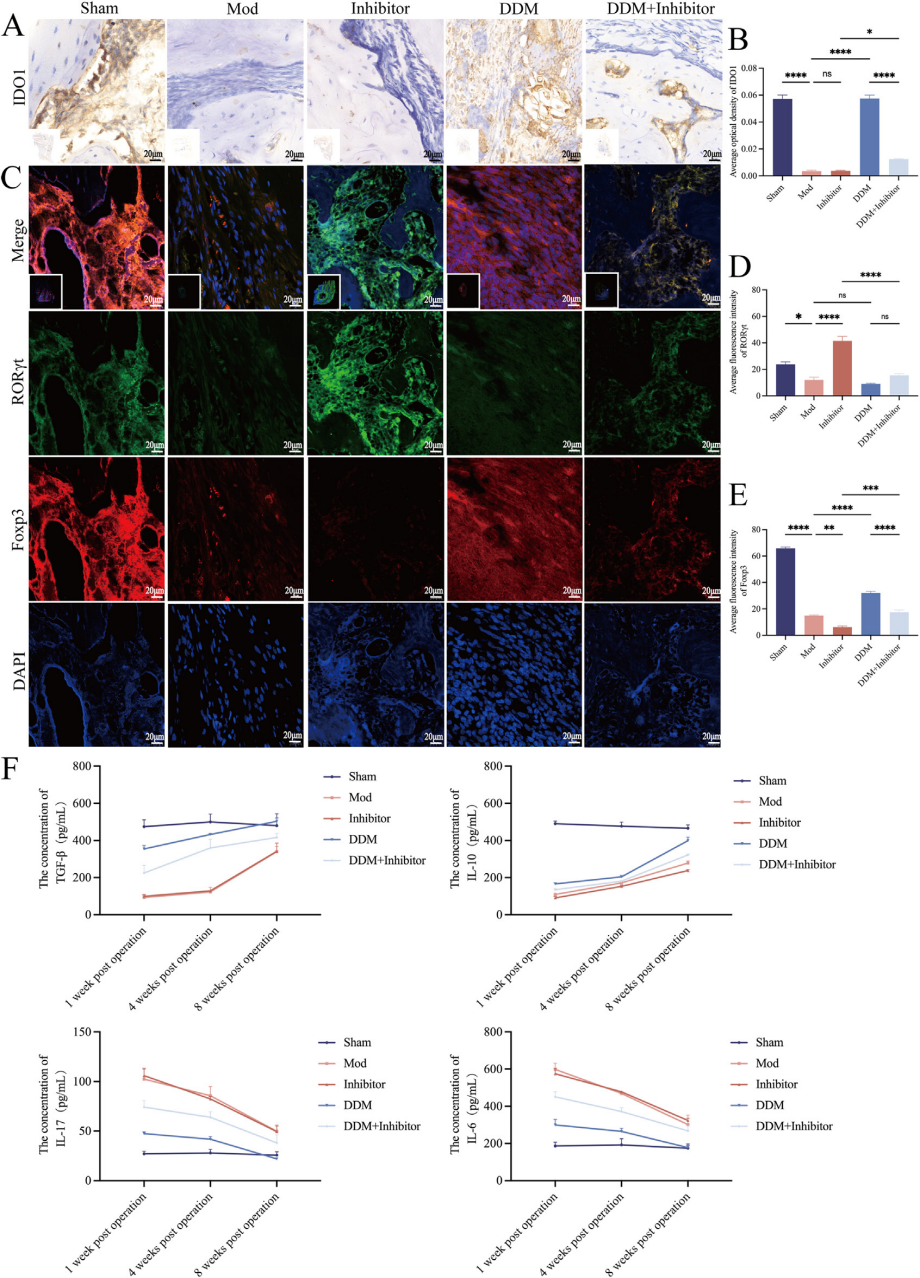
DDM upregulates IDO1 to modulate the Th17/Treg cell balance and improve the inflammatory environment during bone defect repair in New Zealand rabbits. A-B, IHC results show that DDM significantly increased IDO1 expression, which was significantly reduced in the DDM+Inhibitor group. C-E, IF results show that Foxp3 (red) expression significantly increased in the DDM group compared to the Mod group, whereas RORγt (green) remained unchanged. The DDM-induced increase in Foxp3 was suppressed when it was combined with the IDO1 inhibitor. Local image: 400X, 1 cm:20 μm. Full-field image: 4X, 1 cm:2,000 μm. F, ELISA results demonstrated that DDM significantly increased TGF-β and IL-10 and decreased IL-17 and IL-6 gradually over 1, 4 and 8 weeks post-operation, whereas this ability to improve the inflammatory environment was weakened in the DDM+Inhibitor group. Sham, bone exposed without defect creation; Mod, defect without DDM; Inhibitor, defect treated with an IDO1 inhibitor (without DDM); DDM, Defect treated with DDM; DDM+Inhibitor, defect treated with DDM and IDO1 inhibitor.

To verify the hypothesis that the intrinsic mechanism by which DDM promotes bone defect repair—through activation of IDO1 to enhance Treg cell differentiation—tissue samples were collected and sectioned 1 week after surgery. These samples were then examined using IF experiment to assess the expression levels of the RORγt and Foxp3. IF results demonstrated (Figure 7B) that, in contrast to the Mod group, the expression of Foxp3 significantly increased in the DDM group while the influence on RORγt presented no statistical difference. In the group treated with DDM and the IDO1 inhibitor concurrently, the promoting effect of DDM on Foxp3 was inhibited. This indicates that DDM promotes the differentiation of Treg cells via IDO1 to regulate the Th17/Treg balance, exerting nearly no effect on Th17 cells in New Zealand rabbits.

To further confirm the above observation at the functional level and assess the role of DDM in regulating the inflammatory environment *in vivo*, blood samples were collected from the marginal ear vein of rabbits at 1 week, 4 weeks and 8 weeks post-operation. Serum levels of Th17- and Treg-related inflammatory factors were measured using ELISA kits. ELISA results (Figure 7C and Supplementary Figure 3) revealed that cytokine levels in the Sham group remained stable over time, whereas in the other groups, TGF-β and IL-10 gradually increased and IL-6 and IL-17 gradually decreased. At all time points, the levels of anti-inflammatory factors ranked in descending order as follows: Sham group > DDM group > DDM+Inhibitor group > Mod group, with no significant difference between the Mod group and the Inhibitor group. Notably, TGF-β levels in the DDM group at 8 weeks post-operation were slightly higher than those in the Sham group. In contrast, the levels of IL-6 and IL-17 showed an inverse trend. The stable cytokine levels in the Sham group indicated that the surgical procedures (e.g. incision and suture) were performed under aseptic conditions and did not induce a systemic inflammatory response. These results suggest that DDM significantly increased TGF-β and IL-10 and decreased IL-6 and IL-17 gradually over 1, 4 and 8 weeks post-operation, whereas this ability to improve the inflammatory environment was weakened in the DDM+Inhibitor group. Collectively, these findings demonstrate that DDM regulates the Th17/Treg balance by promoting Treg cell differentiation without affecting Th17 cells. *In vivo*, DDM enhances Treg-related anti-inflammatory factors and suppresses Th17-related pro-inflammatory factors.

To clarify the promotional effect of DDM on IDO1, the expression and distribution of IDO1 in the mandibular repair tissue at 8 weeks postoperatively were examined by IHC (Figure 7A). Compared with the Mod group, the expression levels of IDO1 in the DDM group were significantly elevated. After concurrent administration of the IDO1 inhibitor, the expression levels of IDO1 were significantly reduced, indicating that DDM has the capacity to increase the expression level of IDO1. Compared with the Sham group, the expression levels of IDO1 in the Mod group and the inhibitor group were significantly decreased.

*In vivo* experiments conducted in New Zealand rabbits have indicated that the DDM is capable of effectively facilitating the repair of mandibular bone defects in rabbits. The underlying mechanism pertains to regulating the balance of Th17/Treg cells and the expression of related cytokines via IDO1. Simultaneously, DDM significantly increased the levels of TGF-β and IL-10 and reduced the levels of IL-6 and IL-17, thereby optimising the immune environment during the bone defect repair process.

## Discussion

Craniofacial bone defects are commonly caused by infections, trauma or tumours, necessitating bone grafts.^31^, ^32^, ^33^ DDM has emerged as a promising scaffold material for the bone grafts, sparking a growing interest in its modification for applications in both dentin and bone regeneration.^34^^,^^35^ Nevertheless, current research primarily focuses on the osteogenic properties of DDM, leaving its osteoimmunological mechanisms largely unexplored.

Th17/Treg balance has been noticed as one crucial factor in bone regeneration.^12^ Both Th17 and Treg cells differentiate from the same type of naïve CD4^+^ T cells and can interconvert under specific cytokine microenvironments.^36^ Experiments conducted at the cellular, protein, mRNA and functional levels in this study demonstrated that a DDM extract influences the Th17/Treg balance by promoting the differentiation of Treg cells in PBMCs, with minimal impact on Th17 cells.

Our work discovered that IDO1 was a significantly differentially expressed gene affected by the DDM extract in Th17/Treg cells differentiation. It was known that IDO1 is involved in mediating the immunosuppressive effects of Treg cells.^24^ Studies have shown that in patients with rheumatoid arthritis, Treg cells express CTLA-4, thereby exerting immunosuppressive effects, a process closely associated with the enhanced function and upregulated activity of IDO1.^37^ This may be partly attributed to IDO1-mediated tryptophan depletion, which activates GCN2 kinase and inhibits mTOR pathway, thereby suppressing CD8^+^ T-cell proliferation and promoting Treg differentiation.^38^ Tryptophan is essential for the effector functions of T lymphocytes. Tryptophan restriction impairs T-cell proliferation, whereas its catabolites mediate apoptosis and promotes Treg polarization.^39^

Within the co-culture system of naïve CD4^+^ T cells and BMSCs, a DDM extract significantly enhanced the cell viability and osteogenic differentiation capacity of BMSCs. This promoting effect was partially dependent on the immunomodulatory mechanism mediated by DDM. It should be noted that, in the co-culture system of naïve CD4^+^ T cells and BMSCs, the expression level of RANKL was significantly augmented by the DDM extract, and this augmentation effect was attenuated by an IDO1 inhibitor. The expression levels of RANK showed no significant differences among each group. This might be because DDM does not directly act on RANK. Furthermore, osteoblasts can closely regulate the interaction between RANKL and RANK by secreting OPG, thereby influencing the differentiation and activity of osteoclasts. Even if the expression of RANKL increases, if the expression of OPG also increases correspondingly, it may inhibit the activation of RANK by competitively binding to RANKL, thereby preserving the resorption–formation equilibrium.^40^ Therefore, DDM may counteract the influence of increased RANKL by upregulating the expression of OPG, thereby promoting the osteogenic process without significantly altering the expression of RANK.

Based on the findings from early studies, the expression of IDO1 in rheumatoid arthritis synovial fluid^41^ and subsequent research in a collagen-induced arthritis mouse model that revealed that IDO1 deficiency significantly exacerbated the severity of arthritis symptoms and tissue damage, it can be inferred that the presence of IDO1 may alleviate inflammation. In IDO1-deficient mice, this aggravated disease activity was initially associated with a marked increase in pro-inflammatory cytokines, such as IFN-γ and IL-17, produced by T cells in lymph nodes, and later closely linked to increased infiltration of Th1 and Th17 cells in inflamed joints.^42^ These findings reveal that IDO1 plays a key role in suppressing inflammation, particularly Th17-related inflammation, while its inhibition leads to a pronounced inflammatory response. This aligns with the results observed in the rabbit model in the current study. In the sham group, which was in a relatively stable environment with minimal inflammation, higher expression levels of IDO1 and Foxp3 were observed while RORγt expression was lower. However, when the IDO1 inhibitor was administered alone, IF results revealed an increase in the Th17 transcription factor RORγt, and ELISA results demonstrated that the IL-17 levels in the DDM+Inhibitor group were significantly higher than those in the DDM group. These findings further support the notion that IDO1 suppression enhances Th17-mediated inflammation, consistent with earlier observations.

The metabolite of IDO1, kynurenine, can promote the generation and function of Treg cells by activating AhR, thereby enhancing immune tolerance. In inflammatory diseases, the expression of IDO1 helps to maintain immune homeostasis and prevents the onset of autoimmune diseases.^43^ However, in chronic inflammatory and tumour microenvironments, the sustained high expression of IDO1 may lead to immunosuppression, making it difficult to completely eliminate pathogens or tumour cells.^44^ Thus, the role of IDO1 in inflammatory responses is complex and dualistic. During the early stages of inflammation, IDO1 expression helps to suppress inflammatory responses and maintain immune tolerance. However, in chronic inflammation and tumour microenvironments, the persistent high expression of IDO1 may result in immunosuppression, promoting the chronicity of inflammation. The effects of IDO1 depend on the stage of inflammation, cell type and microenvironmental conditions.

This work has disclosed the osteoimmunological role of DDM in regulating the Th17/Treg balance; however, it remains ambiguous which component of DDM plays a key role. Future studies could delve deeper into the specific influences of different physical properties of DDM (such as granular, flaky, blocky and root-like) on the behaviour of immune cells and tissue regeneration. It is acknowledged that DDM can act as a repository for bone tissue repair growth factors, establishing a sustained-release system in local bone defect regions and gradually releasing growth factors such as BMPs, TGF-β, OPN and COL-1.^3^^,^^45^^,^^46^ Future research could further isolate and identify the principal chemical constituents in DDM extracts to clarify which components are most critical for regulating the Th17/Treg balance. Through component analysis and functional validation, the key components with immunomodulatory activity in DDM can be determined.

In summary, we demonstrate that DDM promotes Treg cell differentiation by activating IDO1, thereby influencing the Th17/Treg balance while having minimal impact on Th17 cells. This immunomodulatory mechanism is one of the key reasons for the excellent bone repair outcomes achieved by DDM. It is anticipated that future research will capitalise on the mechanism by which DDM regulates the Th17/Treg cell balance via IDO1 to conduct more targeted bone tissue engineering research, thereby achieving the ideal osteogenic outcome.

## CRediT authorship contribution statement

**Shuyu Zhu:** Conceptualization, Data curation, Formal analysis, Investigation, Methodology, Project administration, Validation, Visualization, Writing – original draft, Writing – review & editing. **Rongkun Chen:** Conceptualization, Writing – review & editing. **Shu Zhang:** Data curation, Formal analysis, Resources, Visualization, Writing – review & editing. **Zhiya Wang:** Investigation, Resources. **Xueyuan Meng:** Investigation. **Peng Ling:** Resources. **Jing Zhou:** Conceptualization, Writing – review & editing, Methodology, Investigation, Supervision. **Zhigang Xie:** Conceptualization, Funding acquisition, Methodology, Project administration, Supervision, Writing – original draft, Writing – review & editing.

## Conflict of interests

None declared

## Appendix Supplementary materials

Image, application 1

Image, application 2

Supplemental Figure 1 demonstrates characterization and multidirectional differentiation potential of the purchased BMSCs. Supplemental Figure 2 shows DDM promotes BSP, Osx, OPN and OPG via IDO1 in New Zealand rabbits. Supplemental Figure 3 includes bar graphs to shows the levels of TGF-β, IL-10, IL-17 and IL-6 across each groups (Sham, Mod, Inhibitor, DDM, DDM+Inhibitor) post-operation. Supplemental Table 1 and Supplemental Table 2 show the reagents and major instruments primarily used in this experiment. Image, application 3

## Footnotes

Supplementary material associated with this article can be found in the online version at doi:10.1016/j.identj.2025.103853.
