# Supplementary material for: Demineralized Dentin Matrix Promotes Bone Regeneration Through IDO1-Mediated Th17/Treg Cell Balance Modulation
Source: Int Dent J. 2025 Sep 4;75(6):103853. doi: 10.1016/j.identj.2025.103853 (PMC12446538; doi:10.1016/j.identj.2025.103853)
Supplement: Supplementary file 1 [file mmc1.docx]

**Supplemental material**

**Characterization and multi-directional differentiation potential of** **BMSCs**

To authenticate the stem cell characteristics of the purchased BMSCs, we employed flow cytometry to detect the specific markers on the surface of BMSCs. The results demonstrated (Supplemental Figure 1A) that the surface stem cell markers CD90 (92.9%) and CD105 (97.58%) of the purchased human BMSCs were positive, while the antigen-presenting cell marker CD45 (6.11%) and hematopoietic stem cell marker CD34 (8.51%) were negative. This indicates that the purchased BMSCs had typical surface marker characteristics of mesenchymal stem cells and met the identification standards of BMSCs.

To detect the osteogenic differentiation capacity of BMSCs, BMSCs were cultured in an osteogenic mediums for 14 days and then subjected to ALP staining. The results displayed blue precipitates (Supplemental Figure 1B), suggesting that BMSCs had successfully differentiated into early-stage osteoblasts with high ALP activity. BMSCs were further cultured in osteogenic mediums for up to 21 days and then underwent ARS staining. The results indicated red calcium nodules (Supplemental Figure 1C), demonstrating that BMSCs had successfully differentiated into mature osteoblasts with high calcium deposition capacity. To examine the adipogenic differentiation capacity of BMSCs, BMSCs were cultured in adipogenic mediums for 21 days and then underwent oil red o staining. The results showed orange-red lipid droplets (Supplemental Figure 1D), indicating that they had successfully differentiated into mature adipocytes with high lipid accumulation capacity. To examine the chondrogenic differentiation capacity of BMSCs, BMSCs were cultured in chondrogenic mediums for 21 days and then underwent alcian blue staining. The results showed blue-stained acidic glycosaminoglycans (Supplemental Figure 1E), indicating that they had successfully differentiated into chondrocytes.

In summary, the purchased BMSCs exhibited typical stem cell surface markers (CD90^+^/CD105^+^, CD45^-^/CD34^-^) and demonstrated multi-directional differentiation potential, including osteogenic, adipogenic, and chondrogenic capacities, confirming their identity as functional BMSCs.


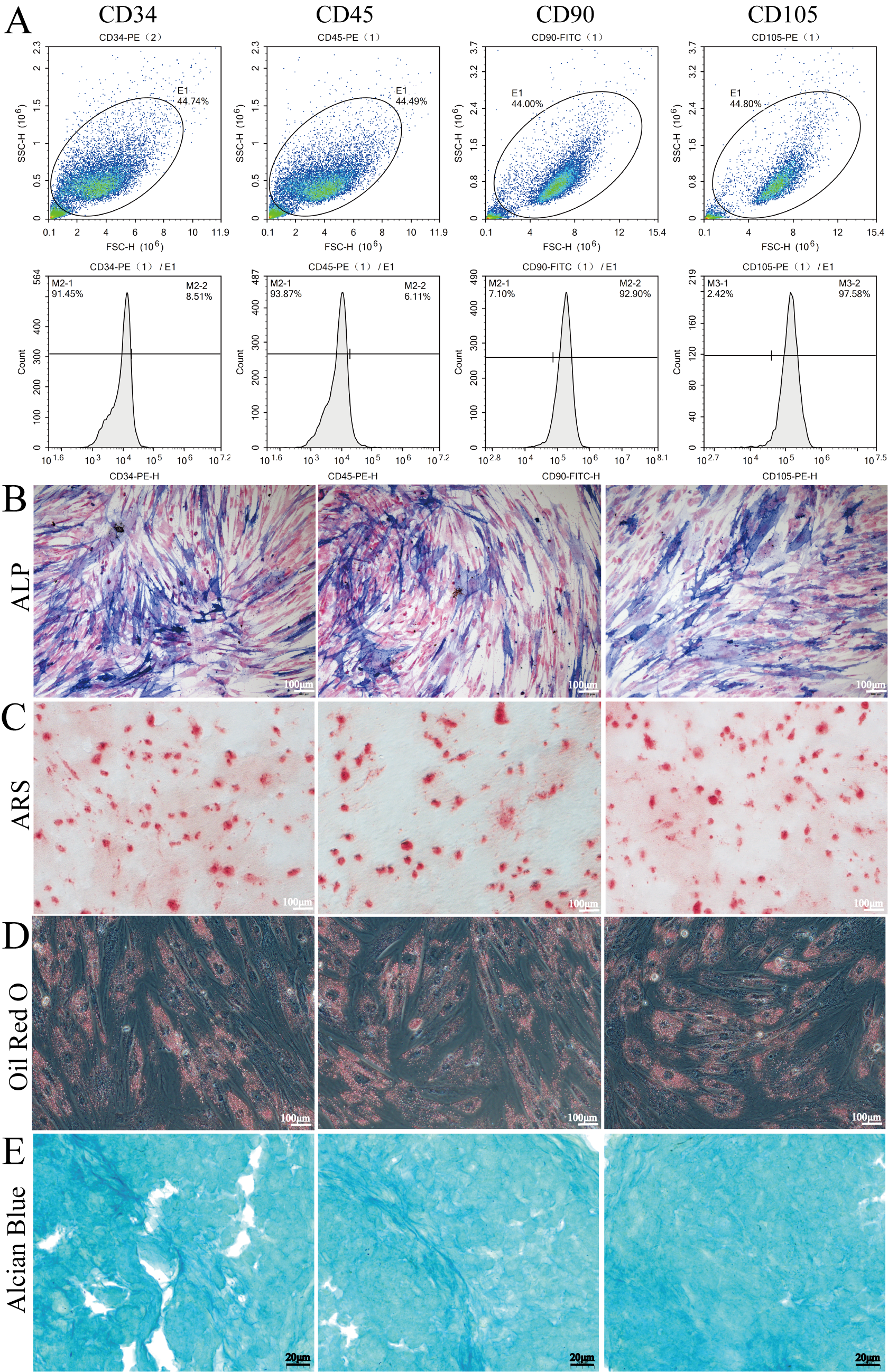


**Supplemental Figure 1. The stem cell characteristics and multi-directional differentiation potential of BMSCs were identified. (A)** Flow cytometry results demonstrated that the surface stem cell markers CD90 (92.9%) and CD105 (97.58%) of the purchased human BMSCs were positive, while the antigen-presenting cell marker CD45 (6.11%) and hematopoietic stem cell marker CD34 (8.51%) were negative. **(B)** Azide-Azide coupling method for ALP staining indicating the blue precipitate after staining. 100X, 1cm:100µm. **(C)** Alizarin red staining indicating the red calcium nodules after staining. 100X, 1cm:100µm. **(D)** Oil red o staining indicating the orange-red lipid droplets after staining. 100X, 1cm:100µm. (E) Alcian blue staining indicating the blue-stained acidic glycosaminoglycans. 400X, 1cm:20µm. All experiments were performed in triplicate.

**Supplemental Figure 2. DDM promotes the expression of osteogenesis-related factors via IDO1 in New Zealand rabbits.** IHC (A-D) and IF (E-H) results demonstrated that compared to the Mod group, the DDM group significantly increased BSP (A-B), Osx (C-D), OPN (E-F), and OPG (G-H) expression, which was reduced by the IDO1 inhibitor. Experimental groups and their Abbreviations: Sham: Bone exposed without defect creation. Mod: Defect without DDM. Inhibitor: Defect treated with IDO1 inhibitor (without DDM). DDM: Defect treated with DDM. DDM+Inhibitor: Defect treated with DDM and IDO1 inhibitor.


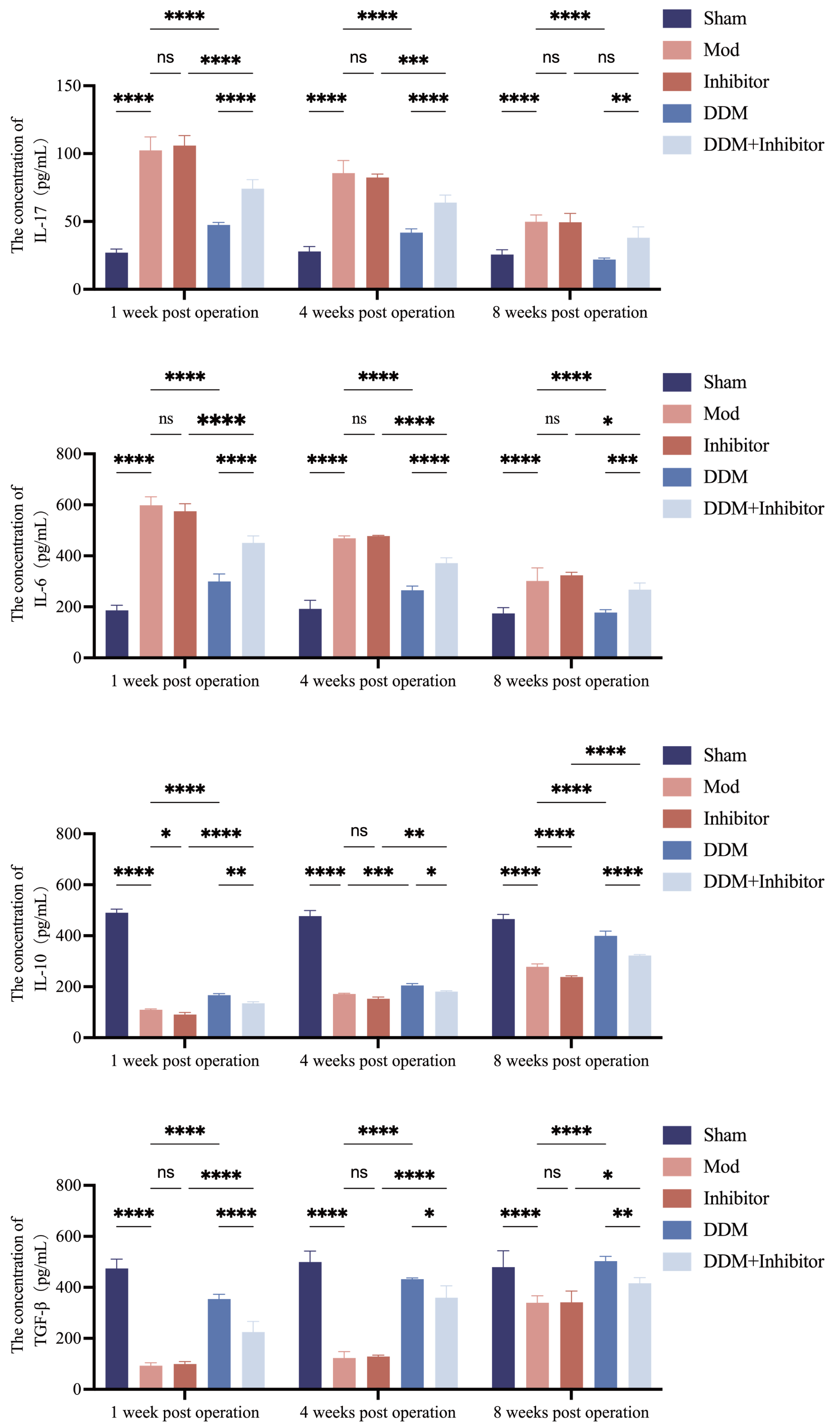


**Supplemental Figure 3. DDM upregulates IDO1 to modulate the Th17/Treg cell balance and improve the inflammatory environment during bone defect repair in New Zealand rabbits.** ELISA results demonstrated that DDM significantly increased TGF-β and IL-10 and decreased IL-17 and IL-6 gradually over 1, 4, and 8 weeks post-operation, while this ability to improve the inflammatory environment was weakened in the DDM+Inhibitor group. Experimental groups and their Abbreviations: Sham: Bone exposed without defect creation. Mod: Defect without DDM. Inhibitor: Defect treated with IDO1 inhibitor (without DDM). DDM: Defect treated with DDM. DDM+Inhibitor: Defect treated with DDM and IDO1 inhibitor.
