# Supplementary material for: Demineralized Dentin Matrix Promotes Bone Regeneration Through IDO1-Mediated Th17/Treg Cell Balance Modulation
Source: Int Dent J. 2025 Sep 4;75(6):103853. doi: 10.1016/j.identj.2025.103853 (PMC12446538; doi:10.1016/j.identj.2025.103853)
Supplement: Supplementary file 2 [file mmc2.docx]

**Supplementary Table 1**：Reagents and Consumables

Supplementary Table 1

| **Name** | **Brand and Origin** |
| --- | --- |
| Demineralized Dentin Matrix (Product Name: Bone Regeneration) | Shenzhen Guangming Chuangbo, China |
| CD25-APC, CD4-FITC, IL-17A-PE, Foxp3-PE | Bioss, China |
| SDS-PAGE Protein Loading Buffer | Biotopped, China |
| TRIZOL Lysis Buffer, Surper ECL Plus Ultra-Sensitive Chemiluminescent Substrate, Prestained Protein Marker | ThermoFisher, USA |
| Foxp3 Primary Antibody (Species Source: Mouse) | Abcam, UK |
| RORγt Primary Antibody (Species Source: Rabbit) | Bioss, China |
| GAPDH Primary Antibody (Species Source: Mouse) | Zhongshan Jinqiao, China |
| Universal Secondary Antibody (HRP-Conjugated Goat Anti-Rabbit IgG) | Sino Biological, China |
| Universal Secondary Antibody (HRP-Conjugated Goat Anti-Mouse IgG) | Abmart, USA |
| Taq Pro Universal SYBR qPCR Master Mix | Nanjing Novozyme, China |
| 96-Well PCR Plate | AXYGEN, USA |
| riboSCRIPT™ mRNA/lncRNA RT-qPCR Starter Kit | Guangzhou RiboBio, China |
| Human IL-10 ELISA Kit, Human IL-17 ELISA Kit, Human IL-6 ELISA Kit, Human TGF-β1 ELISA Kit | Shanghai Enzyme-Linked Biotech, China |
| IDO1 Primary Antibody (Species Source: Rabbit) | Bioss, China |
| Human Naïve CD4+ T Cell Isolation Kit II | STEMCELL, Canada |
| Human Bone Marrow Mesenchymal Stem Cells | Zhongqiao XinZhou, China |
| 1-Methyl-L-Tryptophan | Sigma, USA |
| Osteogenic/Adipogenic/Chondrogenic Induction Medium | Procell, China |
| Alkaline Phosphatase Staining Kit, Alizarin Red S Staining Solution, Alcian Blue Staining Solution, Oil Red O Staining Solution | Solarbio, China |
| CD105-PE, CD34-FITC, CD45-PE, CD90-FITC | ThermoFisher, USA |
| CCK-8 Kit | Biotopped, China |
| RORγt, OPG, Osx, OPN, RUNX2, COL I, BSP, OCN, RANKL, RANK Primary Antibodies (Species Source: Rabbit) | Bioss, China |
| 488-Conjugated Goat Anti-Rabbit IgG, HRP-Conjugated Goat Anti-Rabbit IgG | Sino Biological, China |
| DAPI | Zhongshan, China |
| Citrate Buffer, DAB Chromogenic Substrate Kit | Zhongshan Jinqiao, China |
| EDTA Buffer pH 9.0 | Sino Biological, China |
| Rabbit IL-10 ELISA Kit, Rabbit IL-17 ELISA Kit, Rabbit IL-6 ELISA Kit, Rabbit TGF-β1 ELISA Kit | Shanghai Enzyme-Linked Biotech, China |
| Bilayer Collagen Membrane | Geistlich Bio-Gide, Switzerland |
| Paraformaldehyde | Guangfu, China |
| Paraffin | Surgipath, Germany |
| MX35Premier Plus Superior Blade | ThermoFisher, China |
| Alkaline Phosphatase Staining Solution, Alizarin Red S Staining Solution, Masson's Trichrome Staining Kit | Solarbio, China |
| Hematoxylin | Sigma, USA |
| Eosin Staining Solution | Biotopped, China |
| Histology Pen | Shanghai Xinle Bio, China |
| Histology Cassette | Fuzhou Maxim, China |
| Embedding Cassette | Biosharp, China |
