# Supplementary material for: Demineralized Dentin Matrix Promotes Bone Regeneration Through IDO1-Mediated Th17/Treg Cell Balance Modulation
Source: Int Dent J. 2025 Sep 4;75(6):103853. doi: 10.1016/j.identj.2025.103853 (PMC12446538; doi:10.1016/j.identj.2025.103853)
Supplement: Supplementary file 3 — Supplemental Figure 1 demonstrates characterization and multidirectional differentiation potential of the purchased BMSCs. Supplemental Figure 2 shows DDM promotes BSP, Osx, OPN and OPG via IDO1 in New Zealand rabbits. Supplemental Figure 3 includes bar graphs to shows the levels of TGF-β, IL-10, IL-17 and IL-6 across each groups (Sham, Mod, Inhibitor, DDM, DDM+Inhibitor) post-operation. Supplemental Table 1 and Supplemental Table 2 show the reagents and major instruments primarily used in this experiment. [file mmc3.docx]

**Supplementary Table 2**：Instruments and Equipment

Supplementary Table 2

| **Name** | **Brand and Origin** | **Model** |
| --- | --- | --- |
| -20°C Freezer | Midea, China | BCD-200 |
| 4°C Refrigerator | Zhongxia, China | MTC-700 |
| ECL Imaging System | TianNeng, China | 5200Multi |
| Laminar Flow Cabinet | Haier, China | HCB-1300V |
| Inverted Fluorescence Microscope | OLYMPUS, Japan | DP74 |
| Electrophoresis Power Supply | BAYGENE, China | BG-power300 |
| Quantitative PCR Instrument | Roche, Switzerland | LightCycle 96 |
| Flow Cytometer | ACEA, USA | Novocyte 2060R |
| Microplate Reader | Molecular Devices, USA | MAX190 |
| Vortex Mixer | Sino Biological, China | MV-100 |
| CO2 Incubator | Jingqi, China | CI-191C |
| Upright Fluorescence Microscope | OLYMPUS, Japan | BX53 |
| Whole Slide Scanner | 3DHISTECH, Hungary | Pannoramic MIDI |
| Spectrophotometer | Thermo Scientific, USA | NanoDrop 2000 |
| Agilent 2100 Bioanalyzer | Agilent Technologies, USA | 2100 Bioanalyzer |
| Dental Treatment Unit | KAVO, Germany | INTRAsurg300 plus |
| Bone Harvesting Ring Drill | Bernal Dental, USA | Outer Diameter 8.0mm |
| Rotary Microtome | Leica, Germany | HistoCore BloCu7 |
| Tissue Spreader | Leica, Germany | HI1210 |
| Paraffin Embedding Machine | Leica, Germany | HistoCore ArCadia H |
| Frozen Stage | Leica, Germany | HistoCore ArCadia C |
| Automated Tissue Dehydrator | Wuhan Junjie, China | JT-12S |
| Tissue Spreader and Drier | Wuhan Junjie, China | JK-6 |
| Microwave Oven | Galanz, China | P70D0TL-D4 |
| Micro-CT | Bruker, Germany | SkyScan 1276 |
